# Direct‐Contact Seebeck‐Driven Transverse Magneto‐Thermoelectric Generation in Magnetic/Thermoelectric Bilayers

**DOI:** 10.1002/advs.202308543

**Published:** 2024-03-06

**Authors:** Weinan Zhou, Taisuke Sasaki, Ken‐ichi Uchida, Yuya Sakuraba

**Affiliations:** ^1^ International Center for Young Scientists National Institute for Materials Science Tsukuba 305‐0047 Japan; ^2^ Research Center for Magnetic and Spintronic Materials National Institute for Materials Science Tsukuba 305‐0047 Japan

**Keywords:** anomalous Hall effect, anomalous Nernst effect, Seebeck effect, spin caloritronics, transverse thermoelectric generation

## Abstract

Transverse thermoelectric generation converts temperature gradient in one direction into an electric field perpendicular to that direction and is expected to be a promising alternative in creating simple‐structured thermoelectric modules that can avoid the challenging problems facing traditional Seebeck‐effect‐based modules. Recently, large transverse thermopower has been observed in closed circuits consisting of magnetic and thermoelectric materials, called the Seebeck‐driven transverse magneto‐thermoelectric generation (STTG). However, the closed‐circuit structure complicates its broad applications. Here, STTG is realized in the simplest way to combine magnetic and thermoelectric materials, namely, by stacking a magnetic layer and a thermoelectric layer together to form a bilayer. The transverse thermopower is predicted to vary with changing layer thicknesses and peaks at a much larger value under an optimal thickness ratio. This behavior is verified in the experiment, through a series of samples prepared by depositing Fe–Ga alloy thin films of various thicknesses onto *n*‐type Si substrates. The measured transverse thermopower reaches 15.2 ± 0.4 µV K^−1^, which is a fivefold increase from that of Fe–Ga alloy and much larger than the current room temperature record observed in Weyl semimetal Co_2_MnGa. The findings highlight the potential of combining magnetic and thermoelectric materials for transverse thermoelectric applications.

## Graphical abstract

Simply stacking magnetic and thermoelectric materials can realize a large transverse thermopower. Through formulating the transport properties of a magnetic/thermoelectric bilayer, significant enhancement of transverse thermopower is found under an optimal thickness ratio, which is well reproduced in the experiment. These results shed light on a novel and powerful approach for developing materials for transverse thermoelectric applications.

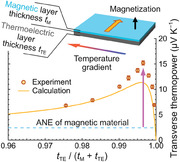

## 1 Introduction

Waste heat generated during various industrial and commercial processes is an enormous yet mostly untapped energy source, and converting it to usable electricity is a crucial component for achieving sustainable global development. The Seebeck effect (SE), which refers to the generation of an electric field parallel to the applied temperature gradient (∇*T*), has long been studied to realize this thermoelectric conversion.^[^ ^1^, ^2^ ^]^ In the recent decade, transverse thermoelectric generation (TTG), with a well‐known example of the anomalous Nernst effect (ANE) observed in magnetic materials, has been attracting increasing interest as an alternative way for such thermoelectric conversion.^[^ ^3^, ^4^, ^5^, ^6^, ^7^, ^8^, ^9^, ^10^, ^11^, ^12^, ^13^, ^14^, ^15^, ^16^, ^17^, ^18^, ^19^, ^20^ ^]^ For ANE, an electric field (**E**) is generated with its direction perpendicular to both ∇*T* and the magnetization (**M**) of the material. The key here is the orthogonal relationship between **E** and ∇*T*, which allows the ANE‐based thermoelectric modules to have a simple two‐dimensional structure made of connecting wires on a surface, in contrast to a complicated 3D structure adopted by the SE‐based modules consisting of alternately placed *p*‐type and *n*‐type semiconductor pillars. This simple structure grants better flexibility and scalability to the ANE‐based modules, and could avoid the efficiency losses and thermal degradation that occurred at the numerous electrical contacts of the SE‐based modules.^[^ ^11^, ^14^, ^16^, ^17^, ^18^, ^20^ ^]^ These advantages have also been exploited for other applications, such as flexible heat flux sensors with low thermal resistance.^[^ ^21^, ^22^, ^23^, ^24^ ^]^ However, the transverse thermopower for ANE is still small compared to the thermopower of SE of thermoelectric materials, and further enhancement is strongly required for creating practical applications.

The thermopower of ANE, anomalous Nernst coefficient *S*_ANE_, is expressed as

(1) $$S_{\mathrm{ANE}}=\rho_{\mathrm{xx}}\alpha_{\mathrm{xy}}-S_{\mathrm{SE}}\frac{\rho_{\mathrm{AHE}}}{\rho_{\mathrm{xx}}}$$

where *ρ*_*xx*_, *α*_*xy*_, *S*_SE_, and *ρ*_AHE_ are the longitudinal resistivity, anomalous Nernst conductivity, thermopower of SE (i.e., Seebeck coefficient), and anomalous Hall resistivity, respectively. The first term on the right‐hand side of Equation (1), *ρ*_*xx*_ *α*_*xy*_ (defined as *S*_I_), is regarded as an intrinsic component of ANE, where *α*_*xy*_ plays a crucial role. Recent studies have shown that large Berry curvature originating from topological electronic structures near the Fermi level leads to large values of *α*_*xy*_, which usually results in large *S*_ANE_ for these magnetic materials as well. The current record‐high *S*_ANE_ > 6 µV K^−1^ at room temperature has been reported for the Weyl semimetal Co_2_MnGa.^[^ ^13^, ^25^, ^26^, ^27^ ^]^ This is a more than an order of magnitude enhancement of *S*_ANE_ from traditional magnetic materials like Fe, Co, and Ni. Exploring magnetic materials with large values of *α_xy_* has thus become a major strategy for achieving large transverse thermopower.^[^ ^28^, ^29^, ^30^, ^31^, ^32^, ^33^, ^34^, ^35^, ^36^, ^37^, ^38^ ^]^ Meanwhile, the second term on the right‐hand side of Equation (1), $-S_{\mathrm{SE}}\frac{\rho_{\mathrm{AHE}}}{\rho_{xx}}$ (defined as *S*_II_), is due to the anomalous Hall effect (AHE) of a magnetic material acting on the longitudinal charge current induced by its SE. Inspired by *S*_II_, recently, a different approach to enhance the transverse thermopower has been proposed and demonstrated: referring to as the Seebeck‐driven transverse magneto‐thermoelectric generation (STTG, see Experimental Section), this approach is based on a closed circuit consisting of a magnetic material and a thermoelectric material with electrical connection only at both ends along the direction of ∇*T*.^[^ ^39^, ^40^, ^41^, ^42^ ^]^ Here, the strong SE of the thermoelectric material generates a much larger longitudinal charge current in the magnetic material, which is then converted to the transverse direction by its AHE, leading to a significant enhancement of transverse thermopower. The value of transverse thermopower is determined by not only the transport properties of the magnetic and thermoelectric materials but also their sizes. Quantitative agreement between experimental demonstrations and the phenomenological formulation has been reported. However, the formation of such a closed circuit requires electrical connection only at the two ends but insulation in between, which could be a complicated structure to be integrated into thermoelectric modules, especially when one tries to reduce the size along the direction of ∇*T* to play to the advantages of ANE‐based modules. A much simpler structure to combine the magnetic and thermoelectric materials and enhance the transverse thermopower would be highly beneficial for the wide adoption of STTG.

**Figure 1:**
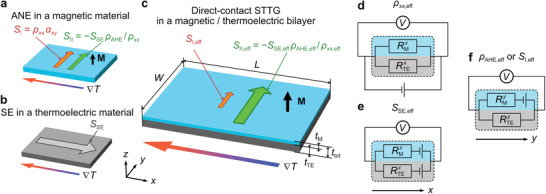
Schematic illustrations of a) ANE in a magnetic material, b) SE in a thermoelectric material, and c) direct‐contact STTG in a magnetic/thermoelectric bilayer. Here, the magnetic material is colored in cyan, while the thermoelectric material is colored in gray. The black arrow represents the direction of **M** of the magnetic material. *L* and *W* in c) are the lengths of the bilayer along the *x*‐axis and *y*‐axis, while *t*_M_, *t*_TE_, and *t*_tot_ are the thicknesses of the magnetic material, the thermoelectric material, and the bilayer, respectively. The equivalent circuits of the magnetic/thermoelectric bilayer for d) the effective longitudinal resistivity (*ρ_xx_*_,eff_), e) the effective Seebeck coefficient (*S*_SE,eff_), and f) the effective anomalous Hall resistivity (*ρ*_AHE,eff_) or the effective intrinsic contribution to the transverse thermopower (*S*_I,eff_).

In this study, we demonstrate STTG‐driven thermopower generation by the simplest way to combine a magnetic material and a thermoelectric material, i.e., stacking a magnetic layer and a thermoelectric layer together to form a bilayer (**Figure** **1**a–c). Unlike the closed circuit used in previous studies of STTG, the magnetic and thermoelectric layers are in direct contact over the entire interface. This means that the fabrication of an insulating layer between the materials and electrical contacts at both ends to connect the materials is no longer needed, leading to a straightforward structure. The lack of electrical contacts also eliminates their potential shunting effect on the transverse thermopower. Therefore, the direct‐contact STTG can be much easier to fabricate and much more versatile to be applied to different length scales and configurations of thermoelectric modules. We model the magnetic/thermoelectric bilayer and derive the expressions for its transport properties, which vary with the thickness of the layers. To experimentally verify the expressions, we characterize a series of samples, which are prepared by depositing Fe–Ga alloy thin films of various thicknesses onto *n*‐type Si substrates. Here, the Fe–Ga films serve as the magnetic material, and the Si substrates serve as the thermoelectric material. The tendencies of transport properties predicted by the expressions are nicely reproduced by the measured results. The transverse thermopower obtained from the sample with optimized layered structure reaches 15.2 ± 0.4 µV K^−1^, which is a fivefold increase from that of the Fe–Ga alloy (*S*_ANE_ = 2.4 ± 0.2 µV K^−1^) and even larger than the predicted maximum of 11.4 µV K^−1^, indicating an additional contribution originated from the interface. Our results shed light on a novel and powerful approach for realizing large transverse thermopower by combining magnetic and thermoelectric materials.

## 2 Results and Discussion

Figure 1c depicts the bilayer model used in this study, where the magnetic layer is colored in cyan and the thermoelectric layer in colored in gray. Both layers have the same length *L* (*W*) along the *x*‐axis (*y*‐axis), while the thicknesses of the magnetic layer, thermoelectric layer, and bilayer are *t*_M_, *t*_TE_, and *t*_tot_, respectively. In order to better facilitate the formulation as well as the exhibition of the results, we also define a fraction $F=\frac{t_{\mathrm{TE}}}{t_{\mathrm{tot}}}$ to represent the thickness ratio, which would take a value between 0 and 1. We consider the transverse thermopower of the bilayer to be parallel to the *y*‐axis, which happens when ∇*T* is applied parallel to the *x*‐axis and **M** of the magnetic material is parallel to the *z*‐axis. For simplicity, we assume that both *t*_M_ and *t*_TE_ are much smaller than *L* and *W*. This means that the change of electrical potential along the *z*‐axis would be much smaller than that in the *x*‐*y* plane, and can be ignored when considering the transport properties in the *x*‐*y* plane. Therefore, we can regard the bilayer as a single hybrid material effectively, and use one parameter to represent the total transverse thermopower ($S_{\mathrm{tot}}^{y}$). However, this limitation on the thicknesses of the layers can be circumvented, which will be discussed later. $S_{\mathrm{tot}}^{y}$ can be formulated by mimicking Equation (1) with the parameters on the right‐hand side replaced by the effective parameters representing the transport properties of the bilayer. These effective parameters can be derived by considering the bilayer as two conductors (representing the magnetic and thermoelectric layers) connected in parallel. Figure 1d shows the equivalent circuit for the effective longitudinal resistivity (*ρ*_*xx*,eff_) of the bilayer along the *x*‐axis. Here, $R_{M}^{x}$ and $R_{\mathrm{TE}}^{x}$ are the resistance of the magnetic and thermoelectric layers along the *x*‐axis, respectively. *ρ*_*xx*,eff_ can be expressed as

(2) $$\rho_{\mathrm{xx},\mathrm{eff}}=\frac{\rho_{M}\rho_{\mathrm{TE}}}{F\rho_{M}+\left(1-F\right)\rho_{\mathrm{TE}}}$$

where *ρ*_M_ and *ρ*_TE_ are the longitudinal resistivity of the magnetic and thermoelectric layers, respectively. *ρ*_*xx*,eff_ is assumed to be isotropic in the *x*‐*y* plane. Figure 1e shows the equivalent circuit for the effective Seebeck coefficient (*S*_SE,eff_) of the bilayer along the *x*‐axis. Here, the two power source symbols represent the electrical potential due to the SE of the magnetic layer (equal to *S*_M_ *L*∇*T*) and the thermoelectric layer (equal to *S*_TE_ *L*∇*T*), with *S*_M_ and *S*_TE_ representing the Seebeck coefficient of the magnetic and thermoelectric materials, respectively. Hence, *S*_SE,eff_ can be expressed as

(3) $$S_{\mathrm{SE},\mathrm{eff}}=\left(S_{\mathrm{TE}}-S_{M}\right)\;\frac{F\rho_{M}}{F\rho_{M}+\left(1-F\right)\rho_{\mathrm{TE}}}+S_{M}$$

It is worth mentioning that Equations (2) and (3) are equivalent to the expressions presented in previous reports that study SE of multilayer systems.^[^ ^43^, ^44^, ^45^ ^]^ As for the effective anomalous Hall resistivity (*ρ*_AHE,eff_) and the effective *S*_I_ (*S*_I,eff_) of the bilayer along the *y*‐axis, their equivalent circuits take a similar form, as shown in Figure 1f. Since there is no transverse effect in a thermoelectric material under zero magnetic field, the power source symbol only exists in the magnetic layer, which equals to $\frac{\rho_{\mathrm{AHE}}UW}{\rho_{M}L}$ (*S*_I_ *W*∇*T*) in case of *ρ*_AHE,eff_ (*S*_I,eff_). Here, *U* is the electrical potential applied to the bilayer along the *x*‐axis. Then, *ρ*_AHE,eff_ can be expressed as

(4) $$\rho_{\mathrm{AHE},\mathrm{eff}}=\frac{\rho_{\mathrm{AHE}}\left(1-F\right)\rho_{\mathrm{TE}}^{2}}{{\left(F\rho_{M}+\left(1-F\right)\rho_{\mathrm{TE}}\right)}^{2}}$$

while *S*_I,eff_ can be expressed as

(5) $$S_{I,\mathrm{eff}}=S_{I}\;\frac{\left(1-F\right)\rho_{\mathrm{TE}}}{F\rho_{M}+\left(1-F\right)\rho_{\mathrm{TE}}}$$

Using these effective parameters described in Equations ((2), (3), (4), (5)), we finally obtain the expression for $S_{\mathrm{tot}}^{y}$ as

(6) $$\begin{array}{ccc} S_{\mathrm{tot}}^{y} & = & S_{I,\mathrm{eff}}-S_{\mathrm{SE},\mathrm{eff}}\frac{\rho_{\mathrm{AHE},\mathrm{eff}}}{\rho_{xx,\mathrm{eff}}}=\frac{\left(1-F\right)\rho_{\mathrm{TE}}}{F\rho_{M}+\left(1-F\right)\rho_{\mathrm{TE}}}\\ & & \left(S_{\mathrm{ANE}}-\frac{F\rho_{\mathrm{AHE}}}{F\rho_{M}+\left(1-F\right)\rho_{\mathrm{TE}}}\left(S_{\mathrm{TE}}-S_{M}\right)\right) \end{array}$$

The part inside the large bracket on the right‐hand side of Equation (6), interestingly, is equivalent to the expression for the total transverse thermopower of a closed circuit, with the second term inside the large bracket being the STTG term.^[^ ^39^ ^]^ Previous studies have shown that in a closed circuit consisting of magnetic and thermoelectric materials, the total transverse thermopower increases with increasing proportion of thickness of the thermoelectric material (which equals to *F* → 1).^[^ ^39^, ^40^, ^41^, ^42^ ^]^ On the other hand, the part outside the large bracket of Equation (6) decreases with increasing *F*, when *F* is between 0 and 1. This indicates that $S_{\mathrm{tot}}^{y}$ studied here would behavior differently comparing to that of a closed circuit.

**Figure 2:**
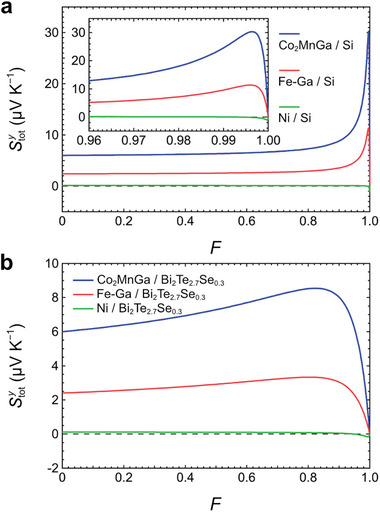
a) $S_{\mathrm{tot}}^{y}$ as a function of *F* calculated using Equation (6) for the bilayers of Co_2_MnGa/Si, Fe–Ga/Si, and Ni/Si. The inset shows the magnified view of the lines in the range of *F* between 0.96 and 1. b) $S_{\mathrm{tot}}^{y}$ as a function of *F* calculated using Equation (6) for the bilayers of Co_2_MnGa/Bi_2_Te_2.7_Se_0.3_, Fe–Ga/Bi_2_Te_2.7_Se_0.3_, and Ni/Bi_2_Te_2.7_Se_0.3_.

In order to illustrate the behavior of $S_{\mathrm{tot}}^{y}$, we calculated $S_{\mathrm{tot}}^{y}$ as a function of *F* using Equation (6) for different combinations of magnetic and thermoelectric materials. Co_2_MnGa, Fe–Ga alloy, and Ni are chosen as the magnetic materials to represent different values in *ρ*_AHE_, while *n*‐type Si and Bi_2_Te_2.7_Se_0.3_ are chosen as the thermoelectric materials to represent different values in *ρ*_TE_ and *S*_TE_. The transport properties of Co_2_MnGa,^[^ ^13^ ^]^ Ni,^[^ ^46^ ^]^ and Bi_2_Te_2.7_Se_0.3_^[^ ^47^ ^]^ are extracted from literature, while the transport properties of Fe–Ga and Si are experimentally measured in this study and will be described in detail later. **Figure** **2**a shows the results when Si (*S*_TE_ = −0.91 mV K^−1^, *ρ*_TE_ = 37.8 mΩ cm) is the thermoelectric material. Here, the value of $S_{\mathrm{tot}}^{y}$ at *F* = 0 corresponds to *S*_ANE_ of the magnetic materials, which changes with increasing *F* and reaches a peak at a certain value of *F*, before it eventually diminishes to 0. When Co_2_MnGa is the magnetic material, the maximum $S_{\mathrm{tot}}^{y}$ can be up to 30 µV K^−1^, a significant enhancement from *S*_ANE_ of Co_2_MnGa (= 6 µV K^−1^), owing to its large *ρ*_AHE_ of 15 µΩ cm. On the other hand, due to the small negative *ρ*_AHE_ of Ni (= −0.045 µΩ cm), the STTG term becomes negative, and $S_{\mathrm{tot}}^{y}$ peaks in the negative region with small magnitude. Another feature here is that the values of *F* for $S_{\mathrm{tot}}^{y}$ to reach the peaks are very close to 1, i.e., a large proportion of thickness of the bilayer is the thermoelectric material. This is due to the fact that *ρ*_TE_ is much larger than *ρ*_M_. In contrast, in the case of Bi_2_Te_2.7_Se_0.3_ (*S*_TE_ = −0.19 mV K^−1^, *ρ*_TE_ = 1.0 mΩ cm) being the thermoelectric material (Figure 2b), the values of *F* for $S_{\mathrm{tot}}^{y}$ to reach the peaks are clearly smaller, since *ρ*_TE_ for Bi_2_Te_2.7_Se_0.3_ is over an order of magnitude smaller than that of Si and much closer to *ρ*_M_. The enhancement in $S_{\mathrm{tot}}^{y}$ is less significant comparing to the results in Figure 2a, due to *S*_TE_ for Bi_2_Te_2.7_Se_0.3_ being much smaller in magnitude than that of Si; still, over 30% enhancement can be achieved for the bilayers containing Co_2_MnGa and Fe–Ga. The calculated results reveal the importance of strong AHE for the magnetic material, strong SE for the thermoelectric material, and an optimal thickness ratio of the bilayer, for realizing a significant enhancement in transverse thermopower.

**Figure 3:**
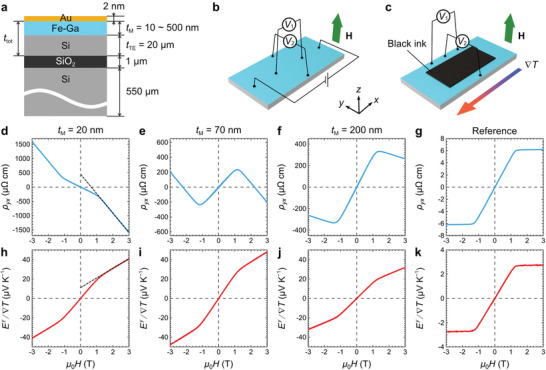
Schematic illustrations of a) a cross‐sectional view of the samples, b) the electrical transport measurement setup, and c) the thermoelectric transport measurement setup. Here, *V*_1_ and *V*_2_ represent two nanovoltmeters measuring the longitudinal and transverse signals, respectively. The black ink layer in c) is for evaluating ∇*T* using an infrared camera. d) *H* dependence of *ρ_yx_* of the sample with *t*_M_ = 20 nm, e) *t*_M_ = 70 nm, f) *t*_M_ = 200 nm, and g) the reference sample for the Fe–Ga alloy. h) *H* dependence of *E^y^* divided by ∇*T* of the sample with *t*_M_ = 20 nm, i) *t*_M_ = 70 nm, j) *t*_M_ = 200 nm, and k) the reference sample. The dashed black lines in d) and h) indicate the linear fitting at high *H* to evaluate *ρ*_AHE,eff_ and $S_{\mathrm{tot}}^{y}$ at zero *H*.

To verify the expressions for the transport properties of the magnetic/thermoelectric bilayer and demonstrate large $S_{\mathrm{tot}}^{y}$ through direct‐contact STTG, we prepared and systematically studied a series of samples with different *F* values. **Figure** **3**a shows a cross‐sectional schematic view of these samples. We used a Silicon‐on‐Insulator (SOI) substrate, which consists of two Sb‐doped *n*‐type Si layers separated by a 1‐µm‐thick SiO_2_ insulator. The top Si layer with *t*_TE_ = 20 µm serves as the thermoelectric layer; the bottom 550‐µm‐thick Si acts as the support for the bilayer. After cleaning the surface of the top Si layer, we deposited the magnetic layer of Fe‐Ga alloy thin films with various thicknesses (*t*_M_ = 10, 20, 40, 70, 100, 150, 200, 350, and 500 nm) at room temperature by magnetron sputtering. The Fe–Ga film serves as the magnetic layer, which is selected for its large *ρ*_AHE_ and minimal thickness dependence of the transport properties, as reported in a previous study.^[^ ^41^ ^]^ The samples were then capped with 2‐nm‐thick Au layers to prevent oxidation (see Experimental Section for details). The composition of the Fe–Ga films was determined to be Fe_70_Ga_30_ by wavelength dispersive X‐ray fluorescence (XRF) analysis. To obtain the transport properties of *n*‐type Si and Fe–Ga, we also carried out measurements on the bare SOI substrate as well as on a reference sample of 100‐nm‐thick Fe‐Ga film deposited on a thermally oxidized Si substrate. The samples were cut into a smaller size of *L* = 10 mm and *W* = 5 mm prior to the transport measurements. Figure 3b,c shows the measurement setups to evaluate the electrical (*ρ_xx_*_,eff_ and *ρ*_AHE,eff_) and thermoelectric (*S*_SE,eff_ and $S_{\mathrm{tot}}^{y}$) transport properties of the samples at room temperature (see Experimental Section for details). From the bare SOI substrate, *ρ*_TE_ was measured to be 37.8 ± 0.8 mΩ cm while *S*_TE_ was measured to be −0.91 ± 0.06 mV K^−1^. Figure 3d–f shows the transverse resistivity (*ρ_yx_*) as a function of out‐of‐plane magnetic field (*H*) for the samples with *t*_M_ = 20, 70, and 200 nm, respectively; while Figure 3h–j shows the transverse electric field (*E^y^*) divided by ∇*T* as a function of *H* for these samples. The results obtained from the Fe‐Ga reference sample are displayed in Figure 3g,k for comparison (see the results from the rest of the samples in Figures S1 and S2, Supporting Information). All the curves show *H*‐odd dependence, as well as saturation of signals above *µ*_0_ *H* ≈ 1.2 T, which corresponds to **M** of Fe–Ga aligned to the direction of *H*. However, the slopes of the curves at high *H* after saturation are different among the samples, especially compared to that of the reference sample being almost horizontal. These slopes are mainly due to the ordinary Hall effect (OHE) and ordinary Nernst effect (ONE) of *n*‐type Si, which manifest themselves as transverse signals changing linearly with increasing *H*. On the other hand, the OHE and ONE of the Fe–Ga film are neglectable compared to their anomalous counterparts, as shown in Figure 3g,k. For samples with small *t*_M_, the *n*‐type Si plays a more important role in determining the overall transport properties, and the OHE and ONE are more prominently displayed in the transverse signals under high *H*; for samples with large *t*_M_, the Fe–Ga film plays a more important role, while the OHE and ONE of *n*‐type Si are shunted by the Fe–Ga. However, since our interests are the anomalous components of the transport properties, we evaluated *ρ*_AHE,eff_ and $S_{\mathrm{tot}}^{y}$ by extrapolating the curves at high *H* after saturation down to zero *H*, as indicated by the dashed black lines in Figure 3d,h. On the other hand, from the results of the Fe–Ga reference sample (Figure 3g,k), *ρ*_AHE_ and *S*_ANE_ are evaluated to be 6.1 ± 0.1 µΩ cm and 2.4 ± 0.2 µV K^−1^, respectively. *ρ*_M_ was measured to be 135 ± 3 µΩ cm while *S*_M_ was measured to be −19.2 ± 1.7 µV K^−1^ under zero *H*. These transport properties of Fe–Ga are consistent with previously reported results.^[^ ^41^, ^48^ ^]^

**Figure 4:**
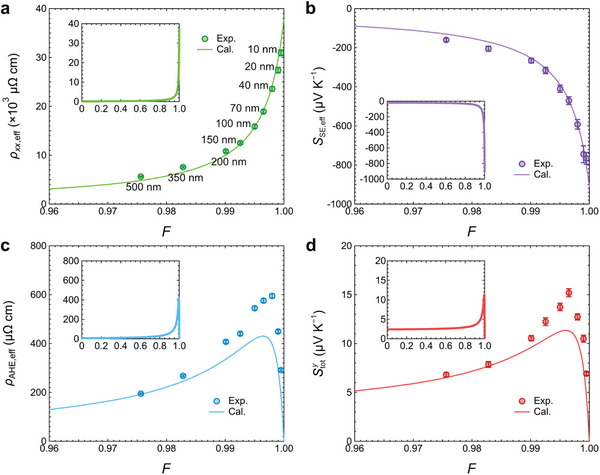
a) *ρ_xx_*_,eff_ as a function of *F*, which is the ratio of *t*_TE_ to *t*_tot_, in the range between 0.96 and 1. The green line is calculated using Equation (2) with the experimentally measured transport properties of Fe‐Ga and *n*‐type Si, while the green data points are measured from the samples with various *t*_M_ as labeled on the points. The inset shows the calculated line in the full range of *F* between 0 and 1. b) *S*_SE,eff_ as a function of *F*. The purple line is calculated using Equation (3), while the purple data points are measured from the samples. The inset shows the calculated line in the full range. c) *ρ*_AHE,eff_ as a function of *F*. The cyan line is calculated using Equation (4), while the cyan data points are measured from the samples. The inset shows the calculated line in the full range. d) $S_{\mathrm{tot}}^{y}$ as a function of *F*. The red line is calculated using Equation (6), while the red data points are measured from the samples. The inset shows the calculated line in the full range. The calculated red line is the same as the red line in Figure 2a.

Using the measured transport properties of *n*‐type Si and Fe–Ga as well as the derived expressions, we calculated *ρ_xx_*_,eff_, *S*_SE,eff_, *ρ*_AHE,eff_, and $S_{\mathrm{tot}}^{y}$ as a function of *F* based on Equations ((2), (3), (4)) and (6), shown as the curves in **Figure** **4**a–d. Here, the insets show the calculated curves in the full range of *F* between 0 and 1; the values at *F* = 0 correspond to the transport properties of Fe–Ga, while the values at *F* = 1 correspond to those of *n*‐type Si. The main figures focus on the range of *F* between 0.96 and 1, where the changes are more prominent. The results measured from the Fe–Ga/Si samples are plotted as data points at the corresponding *F* for comparison. Regarding *ρ_xx_*_,eff_, the experimental result clearly shows a monotonic increase with increasing *F* toward 1, which is in quantitative agreement with the calculated result (Figure 4a). A quantitative agreement can also be seen for *S*_SE,eff_ in Figure 4b, where the values decrease with increasing *F* toward 1 for both the experimental and calculated results. In contrast, *ρ*_AHE,eff_ and $S_{\mathrm{tot}}^{y}$ behave quite differently: the calculated results suggests that both would reach their maximum at certain values of *F*, more specifically, *F* = 0.9964 for *ρ*_AHE,eff_ to peak at 431 µΩ cm while *F* = 0.9960 for $S_{\mathrm{tot}}^{y}$ to peak at 11.4 µV K^−1^ (Figure 4c,d). These tendencies are well reproduced by the experimental results. However, the peak values for both *ρ*_AHE,eff_ and $S_{\mathrm{tot}}^{y}$ are even larger: *ρ*_AHE,eff_ reaches 595 ± 9 µΩ cm at *F* = 0.9980 (*t*_M_ = 40 nm) while $S_{\mathrm{tot}}^{y}$ reaches 15.2 ± 0.4 µV K^−1^ at *F* = 0.9965 (*t*_M_ = 70 nm). One can clearly see that the deviations between the experimental and calculated results for *ρ*_AHE,eff_ and $S_{\mathrm{tot}}^{y}$ are prominent when *F* is close to 1, i.e., for samples with *t*_M_ ≤ 100 nm. This suggests that the Fe–Ga/Si interface could be a factor to these deviations since the interface could have a more significant influence when *t*_M_ is small. It is also worth pointing out that while *ρ*_AHE,eff_ enhanced significantly to almost two orders of magnitude larger than *ρ*_AHE_ at the peak value, the effective anomalous Hall angle of the bilayer monotonically decreases with increasing *F* toward 1 (Figure S3a, Supporting Information). Meanwhile, the agreement between the experimental and calculated results indicates that the change in electronic band structures of Fe–Ga and Si is insignificant due to the formation of a bilayer, with a possible exception for the Fe–Ga/Si interface. Overall, the measured transport properties verify the derived expressions for the magnetic/thermoelectric bilayer, and demonstrate that direct‐contact STTG can indeed significantly enhance the transverse thermopower when magnetic and thermoelectric materials are combined properly.

**Figure 5:**
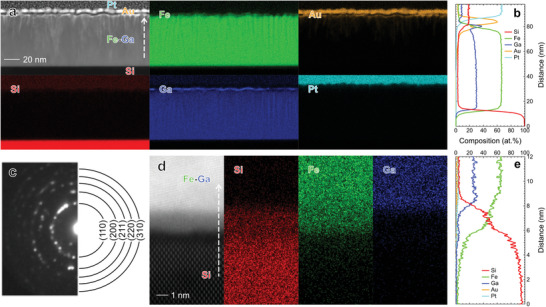
a) Cross‐sectional HAADF‐STEM image of the sample with *t*_M_ = 70 nm, together with the EDS elemental maps of Si, Fe, Ga, Au, and Pt, and b) the corresponding line composition profile along the direction as indicated by the white dashed arrow in a). Pt was deposited during the making of the STEM specimen. c) SAED pattern of the Fe–Ga layer in a). d) HAADF‐STEM image of the same sample focusing on the Fe–Ga/Si interface, together with the EDS elemental maps of Si, Fe, and Ga, and e) the corresponding line composition profile along the direction as indicated by the white dashed arrow in d).

To study the interface between the Fe–Ga and Si layers, we performed scanning transmission electron microscopy (STEM) analysis on the bilayer samples. The results for the sample with *t*_M_ = 70 nm are summarized in **Figure** **5**. The high‐angle annular dark‐field STEM (HAADF‐STEM) image in Figure 5a shows a continuous and flat Fe–Ga layer deposited on the SOI substrate. Based on the energy dispersive X‐ray spectroscopy (EDS) mapping of the relevant elements and the corresponding line composition profile along the out‐of‐plane direction of the sample in Figure 5a,b, most of the Fe–Ga layer has a uniform composition of Fe:Ga = 7:3, which is consistent with the result obtained by XRF analysis, while one can see Ga‐rich regions on a part of the layer's top surface. (Note that the slight increase in Ga composition at the top surface of the Fe–Ga layer was not observed in the sample with *t*_M_ = 20 nm as shown in Figure S4, Supporting Information.) The selected area electron diffraction (SAED) pattern of the Fe–Ga layer (Figure 5c) shows a concentric ring pattern that can be well indexed as a body‐centered cubic structure with no clear superlattice reflections from ordered Fe–Ga phases, suggesting the Ga atoms randomly substitute the Fe lattice. The SAED pattern also shows that the Fe–Ga layer consists of randomly oriented polycrystalline grains. Figure 5d,e shows the high‐resolution HAADF‐STEM image, EDS mapping, and corresponding line composition profile across the Fe–Ga/Si interface. There is an approximately 2‐nm‐thick amorphous‐like layer with dimly imaging contrast consisting of Si and Fe between the brightly imaged crystalline Fe–Ga layer and the darkly imaged Si layer. A similar Fe–Ga/Si interface was observed for the sample with *t*_M_ = 20 nm (Figure S4, Supporting Information). While not considered during the formulation of the expressions, this interfacial layer could contribute positively to *ρ*_AHE,eff_ and $S_{\mathrm{tot}}^{y}$, due to the emergence of spin‐orbit coupling at the interface or ferromagnetic surface state of FeSi.^[^ ^49^, ^50^ ^]^ If the thicknesses of the bilayers are increased, it is possible that the interfacial layer would have a less significant influence on the transport properties, leading to a better agreement between the experimental and calculated results. On the other hand, if the thicknesses of the bilayers are increased to a level that they are comparable to the sample size in the *x*‐*y* plane (*L* and *W*), the assumption for the modeling is no longer satisfied and the derived expressions cannot accurately describe the transverse thermopower of the bilayers. The enhancement of ANE due to the contribution from the interfaces has been previously reported for multilayer structures.^[^ ^51^, ^52^, ^53^ ^]^ Creating multilayer samples of magnetic and thermoelectric materials, which have a certain value of *F* but various numbers of interfaces, could be a helpful way to isolate the effect originating from the interface.

Up to this point, we have been focusing on magnetic/thermoelectric bilayers. However, the derived expressions hold true for multilayer structures consisting of magnetic and thermoelectric materials. In such a case, the thickness of the whole multilayer can be on the same length scale as *W* and *L*, as long as the thicknesses of the individual magnetic and thermoelectric layers are much smaller than *W* and *L*, while *F* would be the ratio of the total thickness of thermoelectric layers to the thickness of the whole multilayer. Equation (6) can then be used to estimate the maximum $S_{\mathrm{tot}}^{y}$ of a certain combination of magnetic and thermoelectric materials, as well as the best thickness ratio to combine the two materials. It is also worth mentioning that $S_{\mathrm{tot}}^{y}$ in direct‐contact STTG is determined by **M** of the magnetic material, the same as in ANE. Therefore, the current magnetic/thermoelectric bilayer can achieve TTG under zero external magnetic field if the magnetic material has anisotropy and is able to maintain **M** perpendicular to both ∇*T* and the generated **E**, such as with *L*1_0_‐FePt^[^ ^39^ ^]^ or SmCo_5_.^[^ ^54^ ^]^

## 3 Conclusion

We have explored the simplest way to combine magnetic and thermoelectric materials, and demonstrated its potential to significantly enhance the transverse thermopower. We considered a model of simply stacking magnetic and thermoelectric layers, and derived the expressions for the transport properties of the hybrid structure, which varies with thickness of the layers. Using the Fe–Ga alloy as the magnetic material and *n*‐type Si as the thermoelectric material, the expressions predict $S_{\mathrm{tot}}^{y}$ to reach a peak value up to 11.4 µV K^−1^ at a certain thickness ratio (*F* = 0.9960), which is a significant enhancement from *S*_ANE_ = 2.4 ± 0.2 µV K^−1^ of the Fe–Ga alloy, and much larger than the current record‐high *S*_ANE_ in a single material (Co_2_MnGa) at room temperature. To experimentally verify this prediction, we prepared a series of samples by depositing Fe–Ga alloy thin films of various thicknesses onto *n*‐type Si substrates, and measured their transport properties. The measured results agree well with all the derived expressions, reproduced the predicted tendency of $S_{\mathrm{tot}}^{y}$ with an even larger value of 15.2 ± 0.4 µV K^−1^ at *F* = 0.9965. The additional contribution to $S_{\mathrm{tot}}^{y}$ may be originated from the Fe–Ga/Si interface. Our results demonstrate that combining magnetic and thermoelectric materials, even in the form as simple as stacking them together into a bilayer, can be a powerful approach for enhancing $S_{\mathrm{tot}}^{y}$. With a great number of studies reporting on different magnetic and thermoelectric materials, the exploration of their combinations with direct‐contact STTG could lead to discoveries of composite materials with excellent properties, which will propel the wide adoption of transverse thermoelectric applications.

## 4 Experimental Section

### Seebeck‐Driven Transverse Magneto‐Thermoelectric Generation

Although in previous studies, the phenomenon has been referred to as Seebeck‐driven transverse thermoelectric generation, to prevent confusion with other types of TTG solely associated with the SE and emphasize the key role of magnetic materials, we added magneto‐ to the name of the phenomenon. The acronym for the phenomenon is retained as STTG.

### Formulation

The effective anomalous Hall angle (tan*θ*_AHE,eff_) of the bilayer can be formulated based on *ρ_xx_*_,eff_ and *ρ*_AHE,eff_, and is given by

(7) $$\tan\theta_{\mathrm{AHE},\mathrm{eff}}=\tan\theta_{\mathrm{AHE}}\;\frac{\left(1-F\right)\rho_{\mathrm{TE}}}{F\rho_{M}+\left(1-F\right)\rho_{\mathrm{TE}}}$$

where $\tan\theta_{\mathrm{AHE}}=\frac{\rho_{\mathrm{AHE}}}{\rho_{M}}$ is the anomalous Hall angle of the magnetic material. Figure S3a (Supporting Information) shows tan*θ*_AHE,eff_ as a function of *F*, where the tendency of results measured from the Fe–Ga/Si samples agrees with the calculation based on Equation (7). With the expressions for $S_{\mathrm{tot}}^{y}$ and *ρ_xx_*_,eff_, we can define the power factor (PF) for TTG of the magnetic/thermoelectric bilayer as

(8)

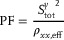




In addition, the effective thermal conductivity (*κ_xx_*_,eff_) of the bilayer in the *x*‐*y* plane can also be derived, by considering it as two thermal conductors (representing the magnetic and thermoelectric layers) connected in parallel. Thus, *κ_xx_*_,eff_ can be expressed as

(9) $$\kappa_{xx,\mathrm{eff}}=\left(1-F\right)\;\kappa_{M}+F\kappa_{\mathrm{TE}}$$

where *κ*_M_ and *κ*_TE_ are the thermal conductivity of the magnetic and thermoelectric materials, respectively. Then, the isothermal figure of merit for TTG (*z_xy_T*) of the bilayer can be expressed as

(10)

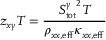

following the definition used by Delves.^[^ ^55^, ^56^ ^]^

### Sample Preparation

The Fe–Ga/Si bilayer samples were prepared by depositing the Fe–Ga films onto the SOI substrates. As shown in Figure 3a, the SOI substrates consist of two Sb‐doped *n*‐type Si layers separated by a 1‐µm‐thick SiO_2_ layer, where the 20‐µm‐thick top Si layer serves as the thermoelectric layer. To ensure a good electrical connection between the Fe–Ga film and the top Si layer, Ar‐ion milling was performed to remove the oxidation layer on top of Si before deposition. Then, without breaking the vacuum, the SOI substrates were transferred to another chamber, where the Fe–Ga films were deposited from a single Fe_65_Ga_35_ alloy target by magnetron sputtering at room temperature. The thicknesses of the Fe–Ga films were varied (*t*_M_ = 10, 20, 40, 70, 100, 150, 200, 350, and 500 nm) by controlling the deposition time. The 2‐nm‐thick Au capping layers were deposited subsequently to prevent oxidation. The Fe–Ga reference sample was prepared by depositing a 100‐nm‐thick Fe–Ga film on a thermally oxidized Si substrate, which was also capped by a 2‐nm‐thick Au capping layer. Before the transport measurements, all the samples were cut into a smaller size of *L* = 10 mm and *W* = 5 mm. All four edges of the samples are either newly cut or covered by sample holders during deposition, to ensure that there is no metallic film on the edges to electrically connect the top and bottom Si layers.

### Transport Measurements

The longitudinal and transverse resistivities of the samples were measured using a Physical Property Measurement System (PPMS) together with its standard resistivity puck. The samples were set on a puck and wire bonding was used to connect the samples to the electrodes on the puck, with the configuration shown in Figure 3b. The electrical connections were fed into external electronics. The power source in Figure 3b represents a Keithley 2401 sourcemeter, while *V*_1_ and *V*_2_ represent two Keithley 2182A nanovoltmeters. *H* was swept along the out‐of‐plane direction by the PPMS during the measurement. The longitudinal and transverse thermopower of the samples was measured using a homemade holder embedded in a multi‐function probe together with the PPMS, as the sample configuration shown in Figure 3c. The homemade holder contains a Peltier module to generate a stable ∇*T* across the sample plane. During the measurement, a constant electrical current (*I* = ±1.0, ±0.8, or ±0.6 A) was applied to the Peltier module, which corresponded to −0.8 K mm^−1^ < ∇*T* < 0.8 K mm^−1^ when the sample reached a steady state. A portion of the surfaces of the samples were coated by black ink having a known emissivity of 0.94, in order to evaluate ∇*T* using an infrared camera. The details of the home‐made holder and the measurement procedure are described in a previous paper.^[^ ^39^ ^]^ The images taken by the infrared camera were also used to evaluate the distances between electrical connections for calculating the parameters. All the transport measurements were carried out at room temperature.

### Scanning Transmission Electron Microscopy Measurement

The electron transparent thin lamella specimens were prepared by standard focused ion beam (FIB) lift‐out technique using an FEI Helios G4 UX system. The STEM‐EDS analysis was performed using an FEI Titan G2 80–200 transmission electron microscope operating at 200 kV.

## Conflict of Interest

The authors declare no conflict of interest.

## Supporting information

Supporting Information

## Data Availability

The data that support the findings of this study are available from the corresponding author upon reasonable request.
