# Supplementary material for: Direct‐Contact Seebeck‐Driven Transverse Magneto‐Thermoelectric Generation in Magnetic/Thermoelectric Bilayers
Source: Adv Sci (Weinh). 2024 Mar 6;11(18):2308543. doi: 10.1002/advs.202308543 (PMC11095155; doi:10.1002/advs.202308543)
Supplement: Supplementary file 1 — Supporting Information [file ADVS-11-2308543-s001.pdf]

ADVANCED SCIENCE
Open Access

Supporting Information

for *Adv. Sci.*, DOI 10.1002/advs.202308543

Direct-Contact Seebeck-Driven Transverse Magneto-Thermoelectric Generation in Magnetic/Thermoelectric Bilayers

Weinan Zhou, Taisuke Sasaki, Ken-ichi Uchida and Yuya Sakuraba*

Supporting Information

Direct-Contact Seebeck-Driven Transverse Magneto-Thermoelectric Generation in Magnetic/Thermoelectric Bilayers

Weinan Zhou, Taisuke Sasaki, Ken-ichi Uchida, and Yuya Sakuraba*

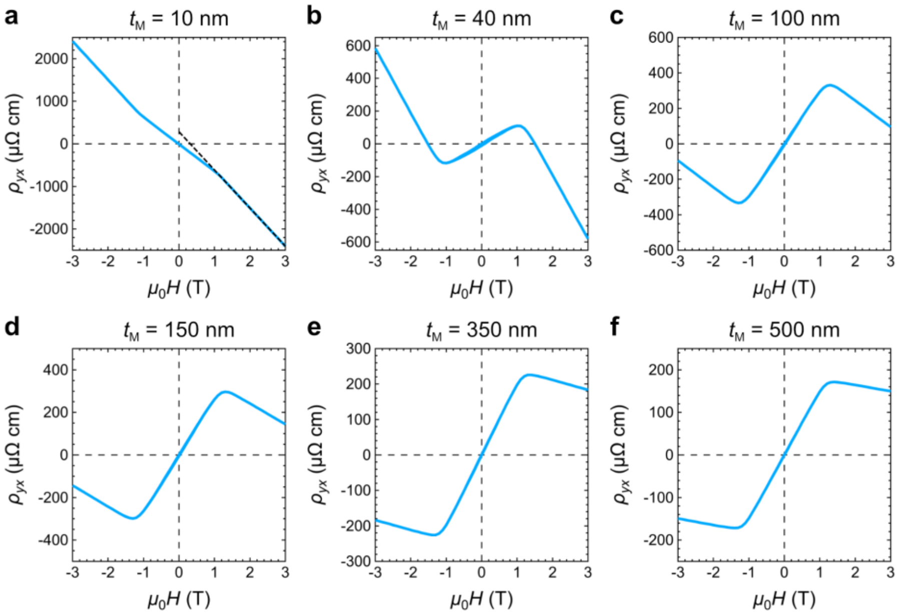

Figure S1. a) H dependence of ρ_{yx} of the sample with t_M = 10 nm, b) t_M = 40 nm, c) t_M = 100 nm, d) t_M = 150 nm, e) t_M = 350 nm, and f) t_M = 500 nm. The dashed black line in a) indicates the linear fitting at high H to evaluate $\rho_{AHE,eff}$ at zero H.

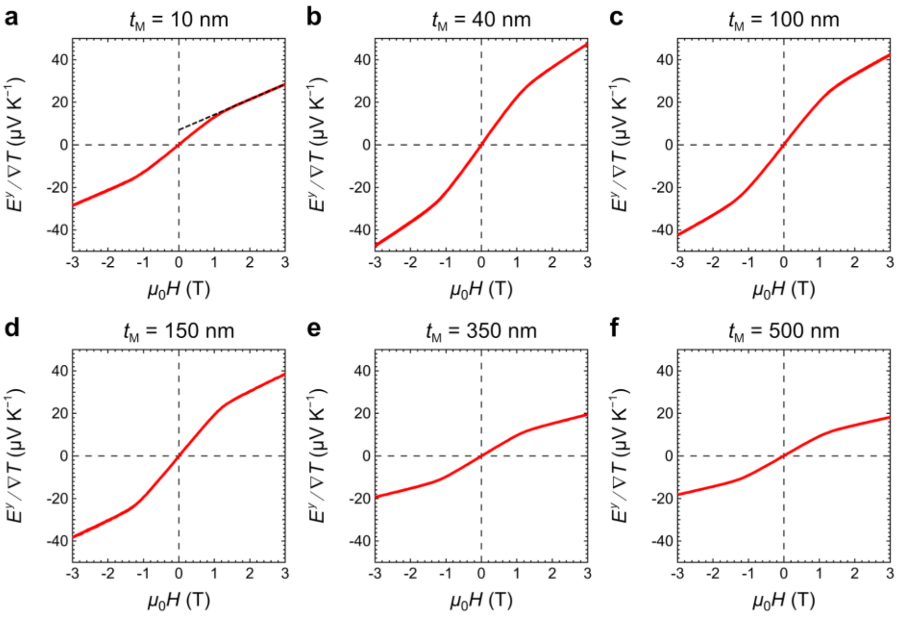

Figure S2. a) H dependence of E^y divided by ∇T of the sample with t_M = 10 nm, b) t_M = 40 nm, c) t_M = 100 nm, d) t_M = 150 nm, e) t_M = 350 nm, and f) t_M = 500 nm. The dashed black line in a) indicates the linear fitting at high H to evaluate S^y_{tot} at zero H.

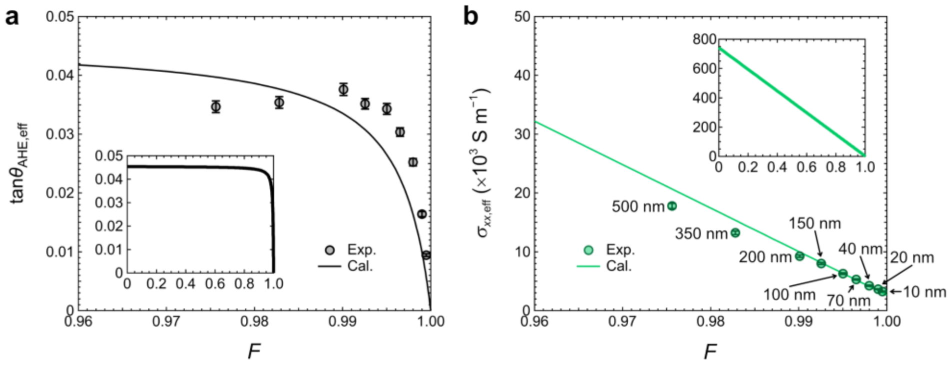

Figure S3. a) $\tan\theta_{\mathrm{AHE,eff}}$ as a function of F, which is the ratio of t_{TE} to t_{tot}, in the range between 0.96 and 1. The black line is calculated using Equation (7) with the experimentally measured transport properties of Fe-Ga and n-type Si, while the black data points are obtained from the measured $\rho_{\mathrm{AHE,eff}}$ and $\rho_{xx,\mathrm{eff}}$ of samples with various t_{M}. The inset shows the calculated line in the full range of F between 0 and 1. b) The effective longitudinal electrical conductivity ($\sigma_{xx,\mathrm{eff}}$) as a function of F. The green line is the calculated using the relationship $\sigma_{xx,\mathrm{eff}} = \frac{1}{\rho_{xx,\mathrm{eff}}} = (1-F)\sigma_{\mathrm{M}} + F\sigma_{\mathrm{TE}}$ ($\sigma_{\mathrm{M}} = \frac{1}{\rho_{\mathrm{M}}}$ and $\sigma_{\mathrm{TE}} = \frac{1}{\rho_{\mathrm{TE}}}$), while the green data points are obtained from the measured results (Figure 4a). The inset shows the calculated line in the full range of F between 0 and 1.

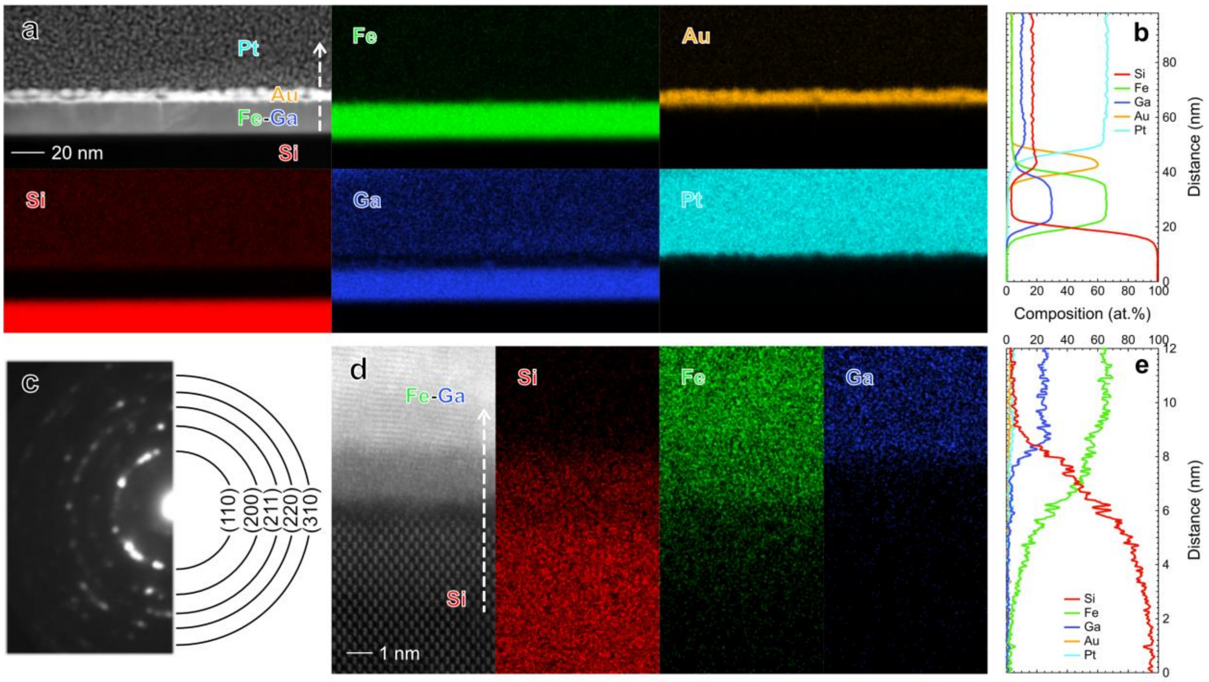

Figure S4. a) HAADF-STEM image of the sample with t_M = 20 nm, together with the EDS elemental maps of Si, Fe, Ga, Au, and Pt, and b) the corresponding line composition profile along the direction as indicated by the white dashed arrow in a). Pt was deposited during the making of the STEM specimen. c) SAED pattern of the Fe-Ga layer in a). d) HAADF-STEM image of the same sample focusing on the Fe-Ga/Si interface, together with the EDS elemental maps of Si, Fe, and Ga, and e) the corresponding line composition profile along the direction as indicated by the white dashed arrow in d).

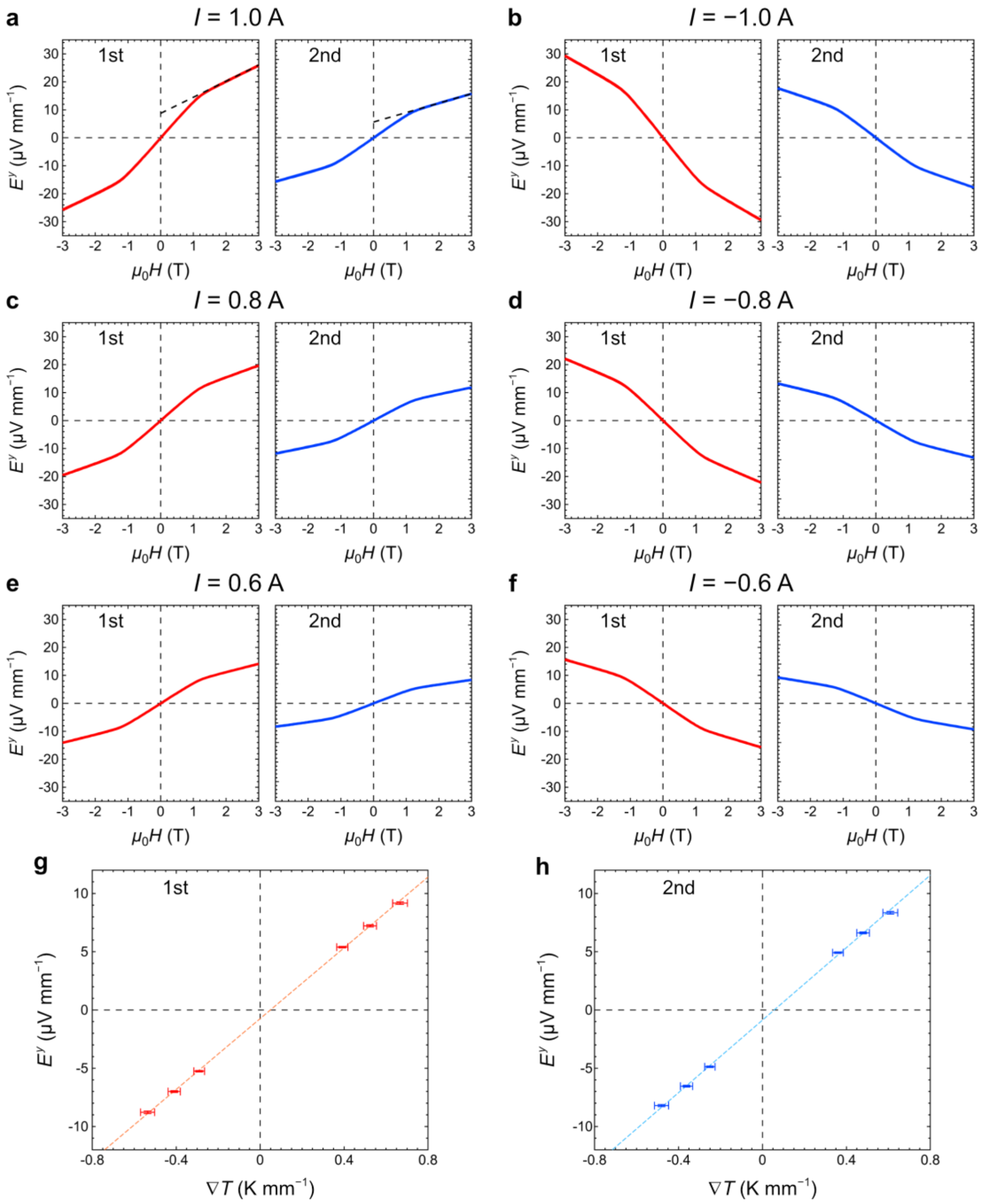

Figure S5. H dependence of E^y of the sample with t_M = 70 nm measured when a) I = 1.0, b) −1.0, c) 0.8, d) −0.8, e) 0.6, f) −0.6 A was applied to the Peltier module. The results labelled 1st are used in the paper, while the remeasured results are labelled 2nd to demonstrate the reproducibility of the measurement. The dashed black lines in a) indicate the linear fitting at high H to evaluate the anomalous component of E^y at zero H. g) The anomalous component of E^y at zero H as a function of the corresponding ∇T for 1st and h) 2nd. The colored dashed lines in g) and h) represent the linear fittings with the error bars taken into consideration, which give S_{tot}^{y} = 15.2±0.4 μV K^{-1} for 1st and S_{tot}^{y} = 15.5±0.5 μV K^{-1} for 2nd.

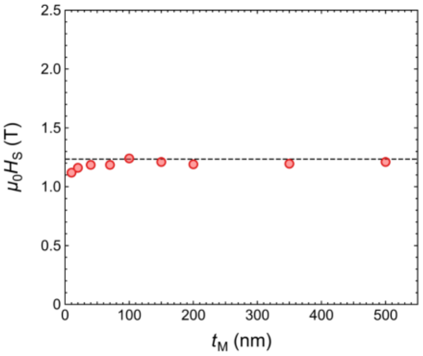

Figure S6. Saturation magnetic field (H_s) of Fe-Ga as a function of t_M. The values are extracted from H dependence of E^y divided by ∇T. The black dashed line indicates the value obtained from the Fe-Ga reference sample.
